# Therapeutic Notes

**Published:** 1901-09

**Authors:** Nelson W. Wilson

**Affiliations:** Buffalo, N. Y.


### PROGRESS IN MEDICAL SCIENCE.

### Therapeutic Notes.

EDITED BY NELSON W. WILSON, M. D., Buffalo, N. Y.

### AN AGREEABLE DIURETIC.

WHEN one needs to give a diuretic the effect may be obtained and in a most pleasant and agreeable manner by giving the following Peabody mixtures, which were printed in the *Medical News*. Two solutions must be used:

| | | | |
|---|---|---|---|
| ℞ No. 1. | Acetate of potash | 26.00 | ʒviss. |
| | Bicarbonate of potash | 12.00 | ʒiii. |
| | Water | 240.00 | ℥viii. |
| Mix. | Label No. 1. | | |
| ℞ No. 2. | Citric acid | 10.00 | ʒiiss. |
| | Water to make | 60.00 | ℥ii. |
| Mix. | Label No. 2. | | |

Directions: To two ounces of water add half an ounce of No. 1 and a teaspoonful of No. 2 and give in 24 hours.

### CALOMEL IN HEMORRHOIDS.

THIS drug is not only curative, but also prevents the plebitis which causes so much pain. For external hemorrhoids give laxatives, and powder with calomel; for internal hemorrhoids use calomel suppositories or an ointment of

| | | | |
|---|---|---|---|
| ℞ | Calomel . . . . . . . . . . . . . . . . | 2.0 | gr. xxx. |
| | Vaseline . . . . . . | | |
| | Lanoline . . . . . . . . . . . . . . . . . . āā | 15.0 | ℥ss. |

Add belladonna or opium if desired. Wash anus with boric-acid water after each defecation.—*Journal de Médecine de Bordeaux*.

### THE USE OF ORTHOFORM.

IN NOSE and throat work, says the *Year-Book of the Nose, Throat and Ear*, the reports of the use of this drug have been almost universally favorable. Brocq, however, has found orthoform sometimes irritating, and capable of exciting hyperemia and pruritus. In one case, a one-to-forty ointment caused swelling and redness lasting three weeks. Asam reports nine cases in which it produced inflammation, and Miodowsky observed a case of moist gangrene following the application of a 5 per cent. ointment.

### IODIDES IN LOCOMOTOR ATAXIA.

ROUX, according to the *Journal of Nervous and Mental Diseases*, in a recent work on the sensory visceral derangements in tabes shows that iodides may irritate the stomach, alter the mucosa. and cause gastritis, with serious consequences. The pain is felt in the lower thorax, especially the left side, and may be severe; it is felt an hour or two after eating, increases gradually, and then disappears before the next meal. Vomiting may ensue, and persistence in the iodide may finally produce typical gastric crises. These are distinguished from true crises by their gradual onset and disappearance, by their development after gastric irritation or at the menses, and by their suppression with a milk diet and discontinuance of the iodide.

### IN PURULENT PLEURISY.

CAPITAN at once aspirates all the fluid possible; if it returns, he again removes it and injects a few drops of camphor and betanaphthol, equal parts, or some peroxide of hydrogen. If the pus then persists, an opening is made under cocaine, by knife or thermocautery, and drainage established. He agrees with Desplats that the operation is simple and that removal of rib is unnecessary. As anesthetic and to avoid cardiac or respiratory difficulties a good injection is 1.0-2.0 c.c. (M xv.-xxx) of

| | | | |
|---|---|---|---|
| ℞ | Hydrochlorate of cocaine | .10 | gr. iss. |
| | Hydrochlorate of morphine | .05 | gr. ¾. |
| | Sulfate of sparteine | .20 | gr. iii. |
| | Sulfate of atropine | .02 | gr. ⅓. |
| | Distilled water to make | 10.00 | ʒiiss. |

This is supplemented by ethyl chlorid spray.

At the first sign of trouble inject

| | | | |
|---|---|---|---|
| ℞ | Sulfate of sparteine | .05 | gr. ¾. |
| | Caffeine | .20 | gr. iii. |
| | Benzoate of soda | .20 | gr. iii. |
| | Distilled water | 1.00 | M xv. |

Repeat if necessary. He lays stress on not operating till the deposits of fibrin have become loosened from the pleura. Normally a certain dulness and indistinct breathing persist after aspiration, and when this disappears above the level of the reaccumulating fluid, it indicates detachment of the fibrin. The results of operation are satisfactory in empyemas due to pneumococcus, streptococcus, or staphylococcus, but not in those of tubercular origin.—*La Med. Mod.*

---

### SCABIES IN CHILDREN.

To avoid the irritating action of the ordinary remedies for itch in children, Léon Perrin rubs the whole body at night with

| | | | |
|---|---|---|---|
| ℞ | Camphor oil of chamom. | 100.0 | ℥iijss. |
| | Ointment of liq. styr. | 20.0 | ʒv. |
| | Balsam of Peru | 5.0 | gr. lxxv. |

The next morning wash with soap and hot water, and powder the skin with starch. If the skin is a little inflamed apply zinc-oxide ointment. The first night the itching ceases, and in eight or ten days the cure is complete. To avoid recurrence all the clothes and bedding must be washed with disinfectants.—*Journal de Médecine de Bordeaux.*

---

### ON THE TREATMENT OF ASTHMA.

Nothing is so likely to induce an attack, say Fowler and Godlee (*Diseases of the Lungs*), as retiring before digestion is finished, therefore the principal meal should be taken at midday. Alcoholic drinks and coffee can rarely be taken safely. To ward off attacks, iodide of potassium, gram 0.3 (gr. v.), three times a day, increased rapidly to 4.0 (ʒi) a day, has been of most service, and to this may be added the ethereal tincture of lobelia, 1.3 (m xx.), or extract of stramonium .015 (gr. $\frac{1}{4}$). Arsenic is worthy of trial, but is of less value. Some patients never retire without burning niter paper, made by saturating blotting-paper with a 1-in-16 solution of potassium nitrate, or smoking a cigarette containing stramonium or some such remedy. As regards locality, every asthmatic is a law to himself; most find that in the smoky atmosphere of a large town they are more free from attacks than elsewhere. If chronic bronchitis is the cause, a warm, dry climate, avoidance of exposure to cold and damp, and cod-liver oil are indicated. When the nervous factor predominates, with slight catarrh or emphysema, a high altitude is usually preferred. Inhalation under pressure of a hot, dense vapor, as at Mont Doré, is often of benefit. When a gouty factor is present, colchicum and alkalies are indicated, and mercury and saline purgatives in constipation and functional derangements of the liver. In some persons these latter are certain precursors of an attack. Morbid conditions of the nasopharynx require local or climatic treatment.

---

### A TALE OF TWO CITIES.

In connection with our comments upon filters, it is only just to remark that again the profession is indebted to the Buffalo Health Department for definite information upon an important point. It has frequently happened before. Inquiry, however, among the oldest Cleveland physicians fails to resurrect a memory of an instance in which the health department of this city has made a contribution to scientific knowledge. This perhaps has not been due so much to the neglect or inefficiency of the incumbent health officer, as to the niggardly policy of the city council in all matters pertaining to the department of health. Health officers have asked for bacteriologists and laboratories, but the city fathers only smiled at the eccentricities of the doctors, while they busied themselves negotiating favors with those from whom the city must purchase merchandise and property. Cleveland may lead Buffalo in population; in sanitary matters it is very far in the rear. It must be added that this is due in part to the fact that for some time Dr. Wende has been the executive head of the Buffalo Health Deparment, and has proved himself to be the most progressive and fearless health officer in any of our cities. Will the time ever come when Cleveland will have a properly equipped health department?—*Cleveland Jour. of Med.*, October, 1900.

---

### LOCOMOTOR ATAXIA.

DR. S. LEDUC, professor of medicine in the School of Medicine, at Nantes (*Gazette Médicale de Nantes*), basing his practice on the theory that the syphilitic origin of locomotor ataxia is scarcely contested today, for a past history of syphilis is found in nearly all ataxics, has injected daily into the muscles of the patient's thigh two grammes—about thirty minims—of the following solution:

| | | |
|---|---|---|
| ℞ | Corrosive sublimate. | |
| | Recrystallized sod. chlor. . . . . . . . . . . . . . . | āā gr. iii. |
| | Aq. dest., m. 300. | |

M. It is said that amelioration was at once manifest. Treatment was continued for periods of three weeks, followed by remissions of fifteen days. Six years from the commencement of the treatment the patient has lost the knee-jerk, and, although some lightning pains persist, he walks well, even at night, and leads a very active life.—*N. Y. Med. Jour.*

---

### THE "NORMAL SALT SOLUTION."

THERE is some variation in the formulæ given by the different writers. Dr. Charles A. L. Reed, in his new Text-book of Gynecology, remarks that Locke has suggested the following formula and reported favorably upon it:

| | | |
|---|---|---|
| ℞ | Calcium chloride . . . . . . . . . . . . . . . . | 3¾ grains. |
| | Potassium chloride . . . . . . . . . . . . . . . . | 1½ " |
| | Sodium chloride . . . . . . . | 2½ drachms. |
| | Sterilized, distilled, or tap water, enough to make . | 1 quart. |

M. The solution may be injected subcutaneously into the intestine, or into a vein.—*N. Y. Med. Jour.*

---

### ANESTHETICS IN CARDIAC DISEASES.

HARE (*American Journal of the Medical Sciences*, August, 1901,) states that very few people, even with grave cardiac and vascular disease, die as the direct effect of an anesthetic. If statistics were looked into it would be found that a larger number of people die at stool or on going upstairs when suffering from disease of the heart than from anesthesia. He thinks that the majority of anesthesia accidents are the results of the shock of the operation and not of the anesthetic.

